# A global cautionary tale: discrimination and violence against trans women worsen despite investments in public resources and improvements in health insurance access and utilization of health care

**DOI:** 10.1186/s12939-022-01632-5

**Published:** 2022-03-03

**Authors:** Sean Arayasirikul, Caitlin Turner, Dillon Trujillo, Sofia L. Sicro, Susan Scheer, Willi McFarland, Erin C. Wilson

**Affiliations:** 1 Trans Research Unit for Equity, Center for Public Health Research, San Francisco Department of Public Health, 25 Van Ness Avenue, Suite 500, San Francisco, CA 94102 USA; 2 Department of Pediatrics, University of California San Francisco, San Francisco, CA USA; 3 Department of Biostatistics and Epidemiology, University of California San Francisco, San Francisco, CA USA

**Keywords:** Transgender women, Discrimination, Violence, Social determinants of health, Health disparities, Health inequities

## Abstract

**Background:**

To determine if improvements in social determinants of health for trans women and decreases in transphobic discrimination and violence occurred over three study periods during which extensive local programs were implemented to specifically address longstanding inequities suffered by the transgender community.

**Methods:**

Interviewer-administered surveys from repeated cross-sectional Transwomen Empowered to Advance Community Health (TEACH) studies in 2010, 2013 and 2016-2017 in San Francisco collected experiences with transphobia violence and discrimination. Respondent-driven sampling was used to obtain a sample of participants who identified as a trans woman.

**Results:**

Violence due to gender identity was prevalent; in each study period, verbal abuse or harassment was reported by over 83% of participants, and physical abuse or harassment was reported by over 56%. Adverse social determinants of health including homelessness, living below the poverty limit, methamphetamine use, depression, PTSD, and anxiety all significantly increased from 2010 to 2016. When testing for trends, housing discrimination and physical violence were both more likely in 2016-2017 compared to the two earlier study periods. Housing discrimination (aOR 1.41, 95% CI 1.00-1.98) and physical violence due to gender identity/presentation (aOR 1.39, 95% CI 1.00-1.92) both significantly increased from 2010 to 2016.

**Conclusion:**

Our findings are particularly alarming during a period when significant public health resources and community-based initiatives specifically for trans women were implemented and could have reasonably led us to expect improvements. Despite these efforts, physical violence and housing discrimination among trans women worsened during the study periods. To ensure future improvements, research and interventions need to shift the focus and burden from trans people to cisgender people who are the perpetuators of anti-trans sentiment, stigma, discrimination and victimization.

## Introduction

The stigma and discrimination faced by transgender women (hereafter abbreviated as trans women) as a result of their gender identity is pervasive and well-documented [1–3]. As a result, trans women experience high prevalence of adverse outcomes including homelessness, poverty, lack of employment opportunities, and violence [4–7]. These in turn result in disproportionately high levels of negative health outcomes and social inequalities, including high HIV incidence and prevalence, substance use, and mental health disorders among trans women [8–10].

The systemic victimization of trans women due to anti-trans discrimination spans high-, middle-, and low-income countries as well as many cultural contexts [11]. A cross-sectional study of trans women in Cambodia across thirteen provinces found that about a third or more participants experienced problems getting a job (31.5%), were sexually abused (39.5%), or forced to have sex (51.6%) due to their gender identity [12]. This study also found that the odds of binge drinking were significantly higher among participants who dropped out of school and thought it was because of their trans identity and those who reported experience of verbal abuse by family members during childhood [12]. Similar findings were found in studies conducted in Latin American and Africa. One study of trans women living in Rio de Janeiro, Brazil found that over 90% of the 345 participants reported having ever experienced discrimination due to their gender identity, and a majority experienced psychological (85.8%) and physical (54.2%) violence due to their gender identity [13]. A cross-sectional study of nine African countries that included Botswana, eSwatini, Ethiopia, Kenya, Lesotho, Malawi, South Africa, Zambia and Zimbabwe found that among 3,798 sexual and gender minority participants, gender minorities experienced significantly higher levels of violence compared to their non-trans, or cisgender sexual minority counterparts; the odds of lifetime violence was highest for transgender women (adjusted odds ratio (OR) 2.51; 95% CI 1.88-3.36) when compared to other gender non-conforming participants (OR 2.42; 95% CI 1.64-3.58), cisgender women (OR 1.94; 95% CI 1.48–2.55) and the reference category of cisgender men [14].

A similar pattern was found for violence occurring in the past year, with the highest rates among transgender women.

In recent years, however, the socio-political environment for transgender and gender nonconforming people has rapidly evolved towards using a human rights framework to focus on health care to address the complex health disparities and social inequities that trans women face. After finding anti-trans violence, stigma, and victimization to be prevalent among trans women in the Dominican Republic, Budhwani et al. identified an “urgent need to implement structural interventions and policies to protect transgender women's health and their human rights” [15]. Similarly, after Wolf and colleagues reviewed the available international guidance and policy statements on the state of trans health, they issued a call to action focused on “offering trans-competent services while supporting existing and/or nascent trans organizations and movements” [16]. Bolstering the systems that serve trans people is part of the critical path in addressing the disproportionate burden of violence and discrimination that trans women experience. We explore this phenomenon in San Francisco, California, USA to contribute to the global dialogue around what effective interventions might be viable for disrupting anti-trans violence and discrimination.

In the United States (U.S.), President Biden’s administration pledged to fight for the ‘safety as well as dignity and justice for all transgender and gender nonconforming people’ [17], and appointed the first transgender federal official confirmed by the U.S. Senate. At the local level in San Francisco, programs specifically designed to address longstanding and profound inequities suffered by the transgender community have been rolled out. For example, in the last five years San Francisco implemented the Gender Health SF Program to extend health care access and gender affirmative health care navigation services for trans patients. The city also created the San Francisco Office of Transgender Initiatives, the first and only trans-led government office in the country working with communities to advance policies, programs, and equity for transgender, gender nonconforming, and LGBTQ citizens. Programs seek to improve health outcomes on the individual level (such as behavioral health interventions to improve HIV prevention and care continuum outcomes), and through structural interventions (such as provision of gender-affirmative health, improving insurance coverage for gender-affirmative procedures, and surgeries and training of clinicians to provide competent care to trans people).

Given programmatic efforts, the changing socio-political environment, and increased visibility of transgender people in recent years, we hypothesized that improvements in the health and well-being of trans women, including increased housing stability, educational opportunities, income levels and mental health outcomes would have occurred over the last several years. To explore this hypothesis, we examined trends in social determinants of health, discrimination, and violence among trans women in San Francisco using data from repeated cross-sectional HIV behavioral surveillance studies.

## Methods

We analyzed data from adult trans women who participated in the Transwomen Empowered to Advance Community Health (TEACH) Studies – TEACH 1 (2010, *n* = 314), TEACH 2 (2013, *n* = 233), and TEACH 3 (2016-2017, *n* = 318); cross-sectional surveys conducted at the San Francisco Department of Public Health. The primary aim of the TEACH studies is to measure HIV prevalence and HIV-related behavioral risk factors among adult trans women in San Francisco using the serial cross-sectional design modeled from the National HIV Behavioral Surveillance Study.

All surveys were interviewer-administered and collected information on socio-demographics, self-reported HIV status, risk behaviors, and mental health characteristics. Detailed study procedures for TEACH 1-3 are provided elsewhere [8, 18, 19]. Briefly, respondent-driven sampling was used to obtain a sample of trans women by enlisting the support of trans women “seeds” who recruited their peers. Seeds were recommended by organizational and community stakeholders such as the SFDPH transgender advisory community during the formative phase of each study cycle. Eligible participants were 18 years or older, a resident of San Francisco, and were assigned male sex at birth but did not identify with the gender typically associated with male sex (e.g. man). Participants were not required to use a specific gender identity category or identify as a trans woman to be eligible. Eligible recruits provided written informed consent, completed an interviewer-administered questionnaire, completed an HIV antibody test, and were asked to refer up to 10 other trans women to the study. The study protocol was reviewed and approved by the Institutional Review Board (IRB) at the University of California, San Francisco (UCSF), approval number: UCSF IRB 19-29460.

Variables were consistently measured and categorized across all three study cycles, with the exception of primary health care access (see below). Each study cycle lasted for six months. For each survey year, participants were grouped by whether or not they reported currently being insured. They were classified as having recently accessed primary health care if they responded “yes” to “Have you seen a doctor, nurse, or other health care provider in the last [6 or 12] months?” This question had a different recall window across surveys: for TEACH 1 and TEACH 2, the recall window was the last 6 months, and for TEACH 3 it was the last 12 months.

Socio-demographic characteristics were described for each survey year and included age (categorized as 18-24, 24-34, 35-39, 40-49, 50 or older), race/ethnicity (non-Hispanic/Latina Black or African American, non-Hispanic/Latina Asian, non-Hispanic/Latina white, Hispanic/Latina, multiple/other races/ethnicities), gender identity (trans woman, female, other), education (less than high school, high school diploma/GED, some college, college graduate and beyond), housing status (owning/renting a house/apartment, living with someone else without paying rent, living in a hotel or SRO, homeless/other), and nativity (born or not born in the U.S.). Annual income was dichotomized at, above, or below the San Francisco poverty limit ($22,600, $22,200, $27,650 for years 2010, 2013, and 2017, respectively) according to the year of the survey [20].

Participants were asked to report their HIV status (negative or positive), substance use (using or not using injection drugs, methamphetamine, crack, cocaine, downers, painkillers, heroin, poppers in the last 12 months), and mental health status (ever or not ever being diagnosed with depression, post-traumatic stress disorder, anxiety).

We evaluated dichotomous (yes vs. no) experiences of discrimination and violence among trans women during each survey year. Three lifetime experiences of discrimination were assessed by asking if participants had ever “experienced trouble getting a job because of [their] gender identity or presentation,” “been denied housing or been evicted because of [their] gender identity or presentation,” or “experienced problems getting health or medical services because of [their] gender identity or presentation.” Lifetime experiences of violence were measured by asking whether participants had ever “been verbally abused or harassed because of [their] gender identity or presentation” or “been physically abused or harassed because of [their] gender identity or presentation.”

Statistical approaches consisted of descriptive and analytic components. We described self-reported socio-demographics, health care access, HIV status, substance use, and mental health diagnosis for each of the three survey samples to assess background trends across survey years. We hypothesized that trends in the aforementioned variables would not necessarily be linear, and therefore conducted chi-squared tests in lieu of Cochran-Armitage trend tests. Additionally, we tested for trends in discrimination and violence across each of the three survey samples using chi-squared tests.

Further, we tested for changes in discrimination and violence after adjusting for differences in sample demographic composition over time. In a multivariable logistic regression model with exposure to discrimination and violence experiences as the outcomes, we included age, gender identity, and race/ethnicity as confounders with an indicator variable for survey year (2016 vs. 2010 and 2013 vs. 2010). Since there were five outcomes measured, we conducted five independent logistic regression models. Finally, we stratified each survey sample by whether participants were Black or African American (non-Hispanic/Latina), white (non-Hispanic/Latina), Hispanic/Latina, or multiple/other races/ethnicities (non-Hispanic/Latina) and evaluated trends in discrimination/violence for each of these subgroups after adjusting for age and gender identity. We used multivariable logistic regressions to model trends for each subgroup. Analyses were conducted in Stata 14 software (StataCorp LP, College Station, TX, USA).

## Results

Table 1 describes socio-demographic characteristics, insurance status and primary health care access, HIV status, substance use, and mental health diagnosis for each survey year. Most participants in each survey were over age 35 and came from racially/ethnically diverse backgrounds. Differences in age (*X*^2^=23.42, *p*<0.01) and race/ethnicity (*X*^2^=16.57, *p*=0.04) were present across years. Compared to prior years, the 2016 sample consisted of a higher percentage of participants between the ages of 25 - 34 (65/318; 20.4%). Fewer participants were 50 years or older in 2010 (80/314; 25.5%). The number of Black/African American participants declined over time (88/314; 28.0% in 2010 vs. 63/233; 27.0% in 2013 vs. 64/314; 20.1% in 2016), as did the number of Asian participants (21/314; 6.7% in 2010 vs. 5/233; 2.1% in 2013 vs. 9/314; 2.8% in 2016). Conversely, the number of Hispanic/Latina participants increased from 2010 (96/314; 30.6%) to 2013 (77/233; 33.0%) to 2016 (118/318; 37.1%). Over half of participants self-reported “female” as their gender, and those who reported genders other than “trans woman” and “female” increased from 2010 (0/314; 0.0%) to 2013 (8/233; 3.4%) to 2016 (16/318; 5.0%). The majority of participants lived below the San Francisco poverty limit and this increased over time (263/314; 83.8% in 2010 vs. 199/233; 85.4% in 2013 vs. 290/314; 91.2% in 2016; *X*^2^=12.82, *p*<0.01). While almost half of the participants reported owning/renting their house/apartment in 2010, only one third owned/rented in 2016. Other differences in housing status were found across years (*X*^2^=16.90, *p*=0.01); namely, homelessness increased by almost 10% from 2010 to 2016.

**Table 1:** Trends in Structural Determinants, HIV, Substance Use and Mental Health among Trans Women, San Francisco, California, 2010-2016

	2010		2013		2016		*X*^2^ test statistic	***P***-value
	(***N***=314)		(***N***=233)		(***N***=318)			
	n	%	n	%	n	%		
Age							23.42	< 0.01
18-24	21	6.7%	17	7.3%	23	7.2%		
25-34	60	19.1%	22	9.4%	65	20.4%		
35-39	46	14.6%	26	11.2%	34	10.7%		
40-49	107	34.1%	84	36.1%	86	27.0%		
50+	80	25.5%	84	36.1%	110	34.6%		
Race/Ethnicity							16.57	0.04
White	52	16.6%	42	18.0%	58	18.2%		
Black	88	28.0%	63	27.0%	64	20.1%		
Latina	96	30.6%	77	33.0%	118	37.1%		
Asian	21	6.7%	5	2.1%	9	2.8%		
Multiple/other	57	18.2%	46	19.7%	69	21.7%		
Gender Identity							15.68	< 0.01
Trans woman	150	47.8%	102	43.8%	143	45.0%		
Female	164	52.2%	123	52.8%	159	50.0%		
Other	0	0.0%	8	3.4%	16	5.0%		
Education							4.88	0.56
Less than High School	84	26.8%	67	28.8%	90	28.3%		
High School Diploma / GED	82	26.1%	71	30.5%	102	32.1%		
Some College	116	36.9%	72	30.9%	99	31.1%		
College Graduate or more	32	10.2%	23	9.9%	27	8.5%		
Annual Income							12.82	< 0.01
At/above poverty limit	51	16.2%	33	14.2%	23	7.2%		
Below poverty limit	263	83.8%	199	85.4%	290	91.2%		
Housing Status							16.90	0.01
Own/Rent	146	46.5%	101	43.3%	102	32.1%		
Live with someone without paying rent	8	2.5%	9	3.9%	13	4.1%		
Hotel or SRO	84	26.8%	64	27.5%	95	29.9%		
Homeless, shelter, or other	76	24.2%	59	25.3%	108	34.0%		
Nativity							3.94	0.14
Not born in US	92	29.3%	51	21.9%	80	25.2%		
Born in US	222	70.7%	182	78.1%	238	74.8%		
Insurance Status							16.37	< 0.01
Uninsured	46	14.6%	28	12.0%	16	5.0%		
Insured	267	85.0%	204	87.6%	299	94.0%		
Accessed Primary Health Care (last 6 months)							11.84	< 0.01
Yes	272	86.6%	203	87.1%	300	94.3%		
No	42	13.4%	29	12.4%	18	5.7%		
HIV Serostatus							1.50	0.47
HIV-negative	175	55.7%	149	63.9%	192	60.4%		
HIV-positive	123	39.2%	84	36.1%	123	38.7%		
Recent Substance Use (last 12 months)								
Injection Drug Use	32	10.2%	33	14.2%	38	11.9%	2.01	0.37
Methamphetamine	42	13.4%	55	23.6%	93	29.2%	7.33	0.03
Crack	63	20.1%	32	13.7%	29	9.1%	3.74	0.15
Cocaine	26	8.3%	33	14.2%	31	9.7%	5.2	0.07
Downers	17	5.4%	14	6.0%	17	5.3%	2.86	0.58
Painkillers	18	5.7%	21	9.0%	17	5.3%	3.43	0.18
Heroin	10	3.2%	15	6.4%	20	6.3%	4.07	0.13
Poppers	19	6.1%	15	6.4%	29	9.1%	2.54	0.28
Lifetime Mental Health Diagnosis								
Depression	143	45.5%	134	57.5%	199	62.6%	20.05	< 0.01
PTSD	59	18.8%	66	28.3%	137	43.1%	45.03	< 0.01
Anxiety	100	31.8%	98	42.1%	180	56.6%	40.18	< 0.01

Insurance coverage and recent primary care visits were reported by most participants and increased significantly from 2010 to 2016. HIV positivity ranged from 36.1% to 39.2% over the survey years with no statistically significant differences across years.

For substance use, only methamphetamine use increased significantly, from 13.4% in 2010 to 29.2% in 2016 (*X*^2^=7.33, *p*=0.03), no statistically significant differences were found for other substances across survey years. Self-reported diagnoses of depression (45.5% in 2010 and 62.6% in 2016; *X*^2^=20.05, *p*<0.01), post-traumatic stress disorder (18.8% in 2010 and 43.1% in 2016; *X*^2^=45.03, *p*<0.01), and anxiety (31.8% in 2010 and 56.6% in 2016; *X*^2^=40.18, *p*<0.01) all significantly increased from 2010 to 2016.

Table 2 describes experiences of discrimination and violence for each survey year. Ever experiencing trouble getting a job due to gender identity/presentation was reported by approximately half of the participants in each survey year (52.5% in 2010; 46.8% in 2013; 50.9% in 2016), and ever experiencing problems getting health/medical services due to gender identity/presentation was reported by approximately one in five participants in each survey year (21.3% in 2010; 25.3% in 2013; 26.4% in 2016). Experiences of housing discrimination based on gender identity decreased from 30.6% in 2010 to 27.5% in 2013, then increased to 37.4% in 2016 (*X*^2^=8.13, *p*=0.02). Experiences of verbal abuse were reported by over three-quarters in each sample (83.1% in 2010; 84.1% in 2013; 85.2% in 2016) and physical abuse was reported by over half in each sample (56.7% in 2010; 63.5% in 2013; 64.2% in 2016).

**Table 2:** Trends in Transphobic Discrimination and Violence among Trans Women, San Francisco, 2010-2016

	**2010**		**2013**		**2016**		*X*^2^ test statistic	***P***-value
	(***N***=314)		(***N***=233)		(***N***=318)			
	n	%	n	%	n	%		
Ever experienced trouble getting a job because of gender identity or presentation							1.86	0.39
Yes	165	52.5%	109	46.8%	162	50.9%		
No	148	47.1%	122	52.4%	149	46.9%		
Ever been denied housing or been evicted because of gender identity or presentation							8.13	0.02
Yes	96	30.6%	64	27.5%	119	37.4%		
No	214	68.2%	167	71.7%	188	59.1%		
Ever experienced problems getting health or medical services because of gender identity or presentation							2.51	0.28
Yes	67	21.3%	59	25.3%	84	26.4%		
No	245	78.0%	173	74.2%	230	72.3%		
Have you ever been verbally abused or harassed because of your gender identity or presentation?							0.52	0.77
Yes	261	83.1%	196	84.1%	271	85.2%		
No	53	16.9%	37	15.9%	47	14.8%		
Have you ever been physically abused or harassed because of your gender identity or presentation?							4.55	0.10
Yes	178	56.7%	148	63.5%	204	64.2%		
No	136	43.3%	85	36.5%	113	35.5%		

After adjusting for age, gender identity, and race/ethnicity, the odds of experiencing housing discrimination due to gender identity/presentation (adjusted odds ratio, aOR 1.41, 95% CI 1.00-1.98, *p*=0.049) and the odds of physical violence due to gender identity/presentation (aOR 1.39, 95% CI 1.00-1.92, *p*=0.049) were both significantly higher in 2016 compared to 2010 (Table 3). Subgroup analysis by race/ethnicity indicated one statistically significant result: in 2016 compared to 2010, the odds of housing discrimination based on gender identity/presentation increased for Black trans women (aOR 2.51, 95% CI 1.16-5.40, *p*=0.02).

**Table 3:** Adjusted Trends in Transphobic Discrimination and Violence among Trans Women, San Francisco, California, 2010-2016

	**2010**	**2013**	**2016**
	aOR (95%CI) ***P***-value	aOR (95%CI) ***P***-value	aOR (95%CI) ***P***-value
Ever experienced trouble getting a job because of gender identity or presentation	Ref	0.83 (0.59 - 1.17) 0.29	0.95 (0.69 - 1.32) 0.77
Ever been denied housing or been evicted because of gender identity or presentation	Ref	0.87 (0.59 - 1.28) 0.48	1.41 (1.00 - 1.98) 0.049
Ever experienced problems getting health or medical services because of gender identity or presentation	Ref	1.24 (0.83 - 1.87) 0.30	1.32 (0.91 - 1.92) 0.14
Ever been verbally abused or harassed because of gender identity or presentation	Ref	1.18 (0.73 - 1.88) 0.51	1.23 (0.79 - 1.92) 0.35
Ever been physically abused or harassed because of gender identity or presentation	Ref	1.35 (0.95 - 1.92) 0.10	1.39 (1.00 - 1.92) 0.049

Notes: Multivariable logistic regression models specify year (2013 vs. 2010 and 2016 vs. 2010) as the exposure and experiences of discrimination/violence as outcomes in 5 independent models adjusting for age, gender identity, and race/ethnicity

## Discussion

Our analysis of trans women over three study periods found that they experienced significant transphobic discrimination and violence in each time period, with no improvement on most measures over time and even worsening with respect to physical violence and housing discrimination. Discrimination in employment was experienced by approximately half of trans women in each study period, and nearly a quarter or more of participants experienced discrimination in both housing and in accessing health or medical services. Violence due to gender identity or presentation was reported by an extremely high proportion of study participants; in each study period, verbal abuse or harassment was reported by over 83% of participants, and physical abuse or harassment was reported by over 56% of participants. Our findings are particularly alarming during a period in San Francisco when significant public health resources and the development of community-based initiatives specifically for trans women were implemented and could have reasonably led us to expect improvements in health and well-being outcomes. In order to ensure future improvements with the safety and health of the trans community, it is time for research and interventions to shift the focus and burden from trans people to a focus on cisgender people who are the perpetuators of the anti-trans sentiment, stigma, discrimination and victimization.

When testing for trends in discrimination and violence across the study periods, we found that housing discrimination and physical violence were both more likely in 2016 compared to the two earlier study periods indicating that the desired and expected improvements over time did not materialize. Additionally, in analyses stratified by race/ethnicity, we found that even though the number of Black trans women sampled in San Francisco decreased during this time period, housing discrimination increased significantly over time among Black trans women and Black trans women were at an increased risk of housing discrimination compared to White trans women. Increasing gentrification in the San Francisco Bay area in recent years has likely contributed to this decrease in the number of Black trans women [21–23] and the housing disparities they face.

In addition to the decline in the number of Black trans women, we found other significant demographic differences and changes over time. We found poor outcomes in each of the social determinants of health measured, with the majority (or more) of participants in each study period reporting extremely high rates of poverty, low educational achievement, and living in hotels, SROs, shelters or being homeless. Again, gentrification overtime has likely exacerbated these outcomes particularly among the most vulnerable [21–23].

The diversity of the trans women community necessitates an intersectional approach to address the complexity of the lived experiences of trans women. The benefits of an intersectionality framework and approach for public health policies have been previously described [24, 25]. In particular, a focus on the specific intersection of race and gender is crucial to understanding both anti-trans and racial discrimination and designing programs and resources that are effective among trans women who are marginalized at the intersection of race and gender [24].

Our study is subject to a number of limitations. First, by measuring outcomes with a series of cross-sectional surveys, we are unable to observe changes for individuals over time. Second, as mentioned above, a number of trans specific programs and resources were implemented in San Francisco during this time frame that may have affected the measured outcomes. Third, these were interviewer-administrated surveys with sensitive topics that could have been subject to social desirability bias. However, this limitation may be mitigated by the use of well-trained, skilled and experienced interviewers, many of whom were peer trans women, who had an established rapport with the study population. Finally, given the intersection of transphobic discrimination, gender discrimination and racial discrimination, without direct measures of gender and racial discrimination, we are not able to assess the impact these would have had in conjunction with the measured transphobic discrimination.

There is some evidence, nonetheless, that local efforts to invest in gender affirmative health care in San Francisco have had some success increasing health care utilization among trans women. Our findings show that the vast majority of trans women reported insurance coverage and primary care access. Moreover, these indicators increased over the study period and could be a result of the local programs specifically for the trans community that were implemented during the study periods. In particular, these programs have strengthened the capacity of the local health care system to increase access to gender affirming health care, mental health services, and HIV testing. Other programs have increased the visibility of trans health and community awareness across local government and to the public. Additionally, research studies have innovated new models of engaging trans women of color in HIV care. In 2011, Transform SF was funded by the San Francisco Department of Public Health (SFDPH) as an HIV testing initiative specifically for the trans community [26]. This study brought together a collaborative of community-based organizations that included the San Francisco Community Health Center, El/la Para TransLatinas, and Instituto Familiar de la Raza to offer education, risk reduction, and incentives to participate in HIV testing. In 2012, the San Francisco Board of Supervisors adopted a resolution to encourage the SFDPH to ensure the provision of medically necessary gender transition-related care. Later that year, the San Francisco Health Commission approved the development of a transgender surgery access program which led to the creation of Gender Health SF, the first initiative of its kind to extend access and peer navigation to gender affirmative health care services for under- and uninsured trans patients in San Francisco [27]. Gender Health SF not only educates patients about how to engage with the public health system to access gender affirming health care and procedures, it also provides training to providers to better serve trans populations on the following topics: cultural humility and collecting sexual orientation and gender identity (SOGI) data, trans cultural competence, pre-surgical assessments for mental health care providers in alignment with WPATH (World Professional Association for Transgender Health) guidelines, and education around procedural and pre- and post-operative care. In 2014, the Transgender Pilot Project (TPP) [28] an MHSA Innovations funded program, managed by the SFDPH, aimed to increase access to mental health services and improve a sense of connectedness among trans individuals, specifically trans women of color. It implemented multiple, culturally informed strategies to support trans women of color and facilitate behavioral health service engagement using a peer support services model, and conducted peer-led outreach and education, a peer-run Transgender Health Fair for linkages to services, and four weekly peer-led, strength-based and resiliency-focused support groups, each with a different theme, that provided a safe space for trans women of color to connect, share their experiences, and connect to services in the community. In 2017, the San Francisco’s Office of Transgender Initiatives (OTI) [29] was founded as the first and only trans-led city government office in the country working with communities to advance policies, programs, and equity for transgender, gender nonconforming, and LGBTQ residents. This same year, the world’s first legally recognized trans cultural district was founded in San Francisco in the historic Tenderloin neighborhood. Both OTI and the Transgender District are committed to raising public awareness and trans visibility through media campaigns, and programming locally and nationally. From 2012-2017, three Health Resources and Services Administration-funded Special Projects of National Significance in the San Francisco Bay Area [30] to engage and retain trans women of color in HIV care. The Butterfly Project utilized peer-led, motivational interviewing, and group-based sessions to increase health promotion behaviors for transgender women of color. The TransAccess Project studied the impact of co-locating of HIV care in a community-based health center, offering drop-in HIV care. The Brandy Martell Project utilized critical race theory to offer trans women of color living with HIV workshops on legal empowerment, transgender rights, and self-defense as well as individualized legal consultation, services, and advocacy in the clinical setting.

These programmatic efforts are being implemented against a continual backdrop of anti-trans discrimination occurring across the world. Internationally, transgender people experience ‘extreme social exclusion that translates into increased vulnerability to HIV, other diseases, including mental health conditions, limited access to education and employment, and loss of opportunities for economic and social advancement’ [31]. Transphobia worldwide often results in frequent episodes of extreme violence towards transgender people and this violence often goes unpunished [31, 32]. Between October 2019 and September 2020, 75 countries and territories worldwide reported murders of trans and gender-diverse people [32]. Additionally, data is not being systematically collected in most countries, and coupled with misgendering by families, authorities, and media, the extent of violence against transgender persons is likely underestimated.

While there is no global reporting system for anti-trans violence, Freedom for All Americans, a bipartisan campaign to secure full nondiscrimination protections for LGBTQ people in the U.S., tracks related anti-transgender legislation [33]. Currently, their legislative tracker lists nearly one hundred bills characterized as anti-transgender covering, for example, restrictions on employment, medical procedures and health access, and participation in sports. Furthermore, 27 U.S. states have no explicit statewide laws protecting prohibiting discrimination based on sexual orientation or gender identity. As a result, in many jurisdictions there is a stark misalignment between efforts to improve the health trajectories of trans women and the political environment. This misalignment and the resulting dissonance can limit how much positive social change is actually possible.

As more time passes without upstream, systemic reforms across multi-sectors of society, including not just medical but also legal, social, and environmental policy reforms, the social position of trans women becomes increasingly burdened by interlocking oppressions and further inequity. Our study found an unfortunate example of this; although health insurance access and utilization of health care has improved among trans women in San Francisco – an important step in creating a society that affirms trans people – these improvements have done little to protect against anti-trans discrimination and violence. The link between violence and discrimination and the right to health and legal gender recognition is well-documented and clear. As Divan, et al, state in their call for action, “Systemic strategies to reduce the violence against trans people need to occur at multiple levels, including making perpetrators accountable, facilitating legal and policy reform that removes criminality, and general advocacy to sensitize the ill-informed about trans issues and concerns” [31].

As the call for global trans rights broadly strengthens, other research has found that despite progress in legal protections much is still unknown. One study examined policies and legal protections for trans people in three major sub-regions in Latin America and the Caribbean, including the Caribbean, Mesoamerica, and South America [34]. It found that anti-trans legislation in the Caribbean and Mesoamerica exacerbated violence against trans people by reinforcing social and cultural acceptance of transphobia and sexist gender stereotypes. The threat of anti-trans violence was so great that it caused many to flee their home countries, and despite legal protections for trans people in South America, the lack of socio-political will to enforce these protections also contributed to the silent sanctioning of anti-trans violence [34].

## Conclusions

The anti-trans stigma, discrimination, and violence observed in our surveys have been confirmed by numerous other studies worldwide [32]. The Human Rights Campaign (HRC) reported that highest number of transgender and gender non-conforming people ever reported were victims of fatal violence in 2020 [35], and they declared that anti-trans violence is epidemic. Experiences of violence and discrimination are often exacerbated by the intersection of racism and sexism. Violence against trans persons of color is particularly high; the HRC report noted that fatal violence disproportionately affected Black trans women. Becerra et al, reported that a majority of their study population of Asian-American transgender persons reported experiencing any form of abuse/violence (66.9%), 52.4% reported abuse/violence from romantic/sexual partner (52.4%), and 29.5% reported harassment/abuse when trying to use a bathroom [36].

The increase in access to and utilization of gender affirmative health care in San Francisco during this time period has not been sufficient to eradicate discrimination and violence against trans women. Despite the investment in public health resources and development of community-based initiatives specifically for trans women, we observed increases in poverty, low educational achievement, and living in hotels, SROs, shelters or being homeless over time with concurrent increasing methamphetamine use, depression, PTSD, and anxiety among study participants over time. Transphobic discrimination in employment, housing and health care access as well as both physical and verbal violence increased to alarmingly high rates. To ensure the safety and health of the global trans community going forward, public health research and interventions need to be realigned to address and stop the perpetuators of violence and discrimination in order to support the most vulnerable populations.

## Data Availability

The data and materials will not be made publicly available because of issues around research ethics and participant confidentiality.
